# Investigating the value of urinary biomarkers in relation to lupus nephritis histopathology: present insights and future prospects

**DOI:** 10.3389/fphar.2024.1421657

**Published:** 2024-07-22

**Authors:** Qianyu Guo, Pengyan Qiao, Juanjuan Wang, Li Zhao, Zhiying Guo, Xiaochen Li, Xiuying Fan, Chong Yu, Liyun Zhang

**Affiliations:** ^1^ Shanxi Bethune Hospital, Shanxi Academy of Medical Sciences, Tongji Shanxi Hospital, Third Hospital of Shanxi Medical University, Taiyuan, China; ^2^ Department of Rheumatology, Shanxi Bethune Hospital, Taiyuan, China; ^3^ Stem Cell Translational Laboratory, Shanxi Bethune Hospital, Taiyuan, China; ^4^ School of Pharmacy, Shanxi Medical University, Taiyuan, China; ^5^ Office of Drug Clinical Trial Institution, Taiyuan, China

**Keywords:** lupus nephritis, urine biomarkers, renal tissue pathology, acute index, chronicity index

## Abstract

Lupus nephritis (LN), a leading cause of death in Systemic Lupus Erythematosus (SLE) patients, presents significant diagnostic and prognostic challenges. Although renal pathology offers critical insights regarding the diagnosis, classification, and therapy for LN, its clinical utility is constrained by the invasive nature and limited reproducibility of renal biopsies. Moreover, the continuous monitoring of renal pathological changes through repeated biopsies is impractical. Consequently, there is a growing interest in exploring urine as a non-invasive, easily accessible, and dynamic “liquid biopsy” alternative to guide clinical management. This paper examines novel urinary biomarkers from a renal pathology perspective, encompassing cellular components, cytokines, adhesion molecules, auto-antibodies, soluble leukocyte markers, light chain fragments, proteins, small-molecule peptides, metabolomics, urinary exosomes, and ribonucleic acids. We also discuss the application of combined models comprising multiple biomarkers in assessing lupus activity. These innovative biomarkers and models offer insights into LN disease activity, acute and chronic renal indices, fibrosis, thrombotic microangiopathy, podocyte injury, and other pathological changes, potentially improving the diagnosis, management, and prognosis of LN. These urinary biomarkers or combined models may serve as viable alternatives to traditional renal pathology, potentially revolutionizing the method for future LN diagnosis and observation.

## 1 Background

The organ most commonly impacted by SLE is the kidney, with LN being the primary cause of mortality among SLE patients (Cervera et al., 2015; Alforaih et al., 2022).Despite advancements in LN treatment, about 35% of patients experience relapse, and 5%–20% advance to end-stage renal disease (ESRD) (Tektonidou et al., 2016).

Renalbiopsy is the gold standard for diagnosing LN, distinguishing pathological subtypes, determining acute and chronic lesions, and guiding further treatment. According to the recommendations of EULAR/ERA-EDTA, in cases where there is no response or recurrence to immunosuppressive therapy, repeat kidney biopsy should be considered (Fanouriakis et al., 2019). Repeated biopsy can help predict kidney prognosis and guide treatment (Moroni et al., 2021).However, due to the invasiveness and poorreproducibility of renal biopsy, repeated biopsy or continuous monitoring of renal pathological changes is actually not feasible.Moreover, current pathological staging for LN does not fully encompass all manifestations, such as thrombotic microangiopathy, vascular inflammation, podocyte injury, and renal tubulointerstitial lesions, which are crucial for informing treatment strategies and prognosis (Yu et al., 2017). This underscores the necessity for developing non-invasive, convenient biomarkers that can dynamically monitor renal severity and activity in LN, forecast renal prognosis, and track therapy responses and disease progression.

Urine samples, compared to other biological sources like tissue or serum, offer a non-invasive, frequently monitorable, and easily collectible, transportable, and storable option. Significantly, being a kidney derivative, urine can directly reflect the organ’s pathological state. Thus, urine biomarkers, also known as “liquid biopsies,” emerge as a promising diagnostic and prognostic tool (Morell et al., 2021).

This article reviews the last decade’s literature on lupus nephropathy and urinary biological markers. It aims to assess the significance of urinary biomarkers from the histopathology perspective of LN. The review integrates invasive histopathology with non-invasive urinary biomarkers and also concentrates on urinary pathological changes not classified by renal histopathology, offering new insights for clinicians.

## 2 Histopathological changes of LN

**FIGURE 1:**
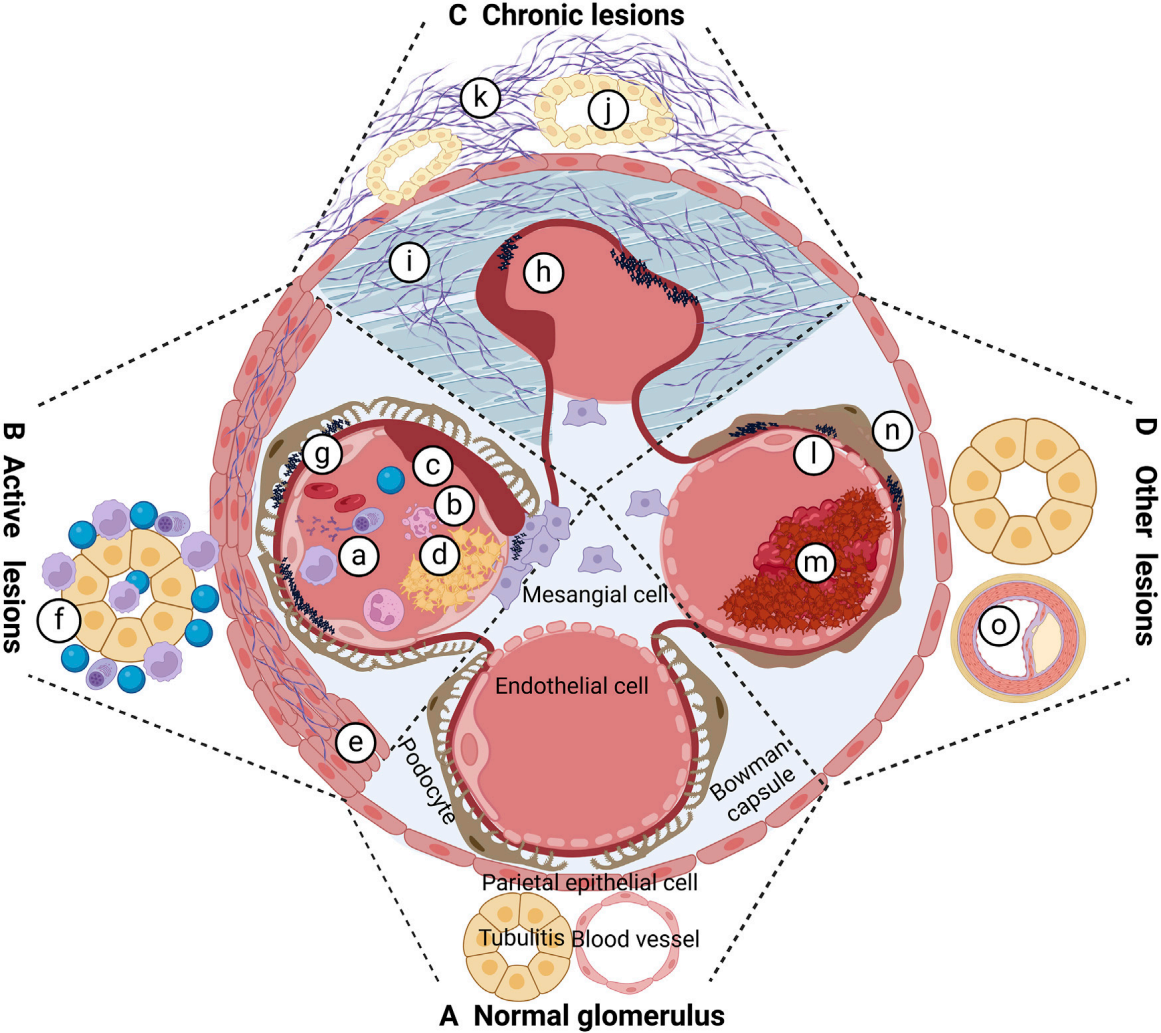
Pathological changes of LN. **(A)** Proliferation of cells in capillaries; **(B)** neutrophil nucleolysis; **(C)** cellulosic necrosis; **(D)** hyaline denature-like deposition, platinum ear and or hyaline thrombus; e cellular crescent and or cellular crescent and or fibro cellular crescent; f interstitial inflammatory cells infiltrated; g immune complex and deposited in different parts; h fiber crescent formation; i Glomerular fibrosis; j tubular atrophy; k interstitial fibrosis; l TMA; m intravascular microthrombus formation; n podocyte damage; o atherosclerotic lesions.

The pathological staging of LN was initially established in the 1960s and has undergone multiple revisions. The 2003 International Society of Nephrology/Renal Pathology Society (ISN/RPS) classification of LN has gained wide acceptance, demonstrating high intra- and inter-observer reliability in guiding treatment strategies and providing prognostic insights (Weening et al., 2004). Nonetheless, this classification does not encompass all pathologic alterations, such as lupus podocytopathy, glomerular collapse, thrombotic microangiopathy (TMA), and tubulointerstitialand vascular abnormalities (Yu et al., 2017; Wilhelmus et al., 2015).The 2018 International Renal Pathology Working Group introduced updates to these overlooked pathologies, including renal tubular interstitial, vascular, and podocyte lesions (Bajema et al., 2018).Furthermore, a modified National Institutes of Health (NIH) scoring system for longevity and activity was adopted to assess acute and persistent lesions in LN, offering more analytical and prognostic utility than the 2003 ISN/RPS criteria (Krassanairawiwong et al., 2021; Umeda et al., 2020; Tao et al., 2020). Figure 1 illustrates the acute and chronic pathological changes observed in LN.

## 3 Potential significance of urinary biomarkers from the LN pathology perspective

**FIGURE 2:**
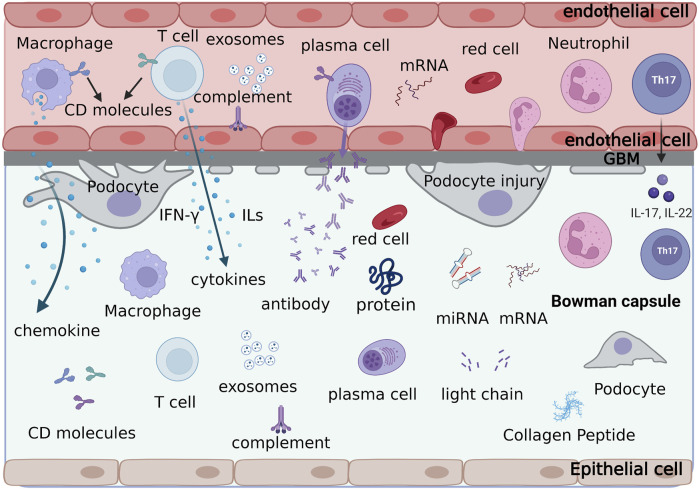
The formation process and types of urine biomarkers. Various cells, cytokines, adhesion molecules, antibodies, light chain fragments, soluble leukocyte markers, proteins and small molecule peptides, metabolomics, exosomes, and ribonucleic acids in the glomerular capillaries are discharged through the damaged glomerular filtration membrane into the Bowman’s capsule, forming the urinary biological markers of lupus nephritis.

Urinary biomarkers can indicate various cellular processes occurring in the glomerulus or tubules, with their levels fluctuating due to inflammation, fibrosis, or active lesions. Urine serves as an ideal biomarker source owing to its easy collection, non-invasive nature, and absence of active proteases, which prevent biomarker degradation (Havanapan and Thongboonkerd, 2009). Figure 2 displays the different types of urine biomarkers.

### 3.1 Cell components

A pivotal study employing single-cell RNA sequencing analyzed kidney samples from LN patients, identifying 21 leukocyte sub-populations during active disease. These included various myeloid, T-cell, natural killer, and B-cell populations. Remarkably, this research revealed a strong correlation between the immune cells’ gene expression in kidney and urine, suggesting urine specimens could serve as an alternative to kidney biopsies for biological information (The Accelerating Medicines Partnership in SLE network et al., 2019).

Urine has been found to have a higher concentration ofinflammatory cells in LN patients. Notably, compared to non-LN-SLE patients, LN patients have higher concentrations of T cells in their urine, particularly CD4^+^T cells (Scott et al., 2016). A study indicated that, in comparison with controls and other chronic nephritis groups, CD4^+^T cells in peripheral blood mono-nuclear cells (PBMC) of LN patients lowered following 6 months of therapy. Conversely, urinary Th17 cells increased gradually. There was a negative correlation between urinary Th17 cells and LN severity, with higher counts of these cells in patients with nonproliferative LN than in those with proliferative LN (Mesquita et al., 2017).

Another study found a significant increase in Ig-secreting Plasmablasts, B cells, or plasma cells (PB/PC) in the urine of 41 LN patients compared to 28 non-LN-SLE patients. Most LN patients with urinary PB/PC exhibited proliferative nephritis and significant renal function loss or had progressed to ESRD. These results imply that urine PB/PC may serve as a biomarker for identifying proliferative LN and patients at risk of progressing to ESRD (Scott et al., 2016).

Using flow cytometry on urine samples from LN patients undergoing renal biopsy, researchers discovered that CD11c^+^ macrophages were significantly more numerous in proliferative LN and correlated substantially with chronic indices such as interstitial fibrosis and tubular atrophy. This suggests that urinary CD11c^+^ macrophage levels are closely linked with chronic pathological changes and renal response in LN, presenting a novel biomarker for the disease (Kim et al., 2020).

The most prevalent cell type in the urine of LN patients was found to be CD14^+^ cells, as shown by a long-term case-control research included 30 LN patients, 30 SLE patients without LN, and 20 healthy controls. Class IV LN had a significantly greater urine CD14^+^ cell count than class III LN, suggesting that it may be used as a biomarker to distinguish between distinct proliferative LN types (Abdelati et al., 2021).

The presence of urinary podocytes and renin levels in children with LN is significantly higher than in other groups. A comparative study revealed that these levels in the focal segmental glomerulosclerosis group were markedly elevated compared to those in minimal change disease groups and mesangial proliferative glomerulonephritis (Wang et al., 2015). Furthermore, severe proteinuria cases showed a notable rise in both the number of podocytes and renal protein expression compared to mild proteinuria cases. In adult LN research, urinary podocyte quantification was substantially greater in the LN group as compared to the control group, with significantly greater levels in individuals with type III, IV, and V LN, particularly in type IV. This correlation with disease activity indicates that urinary podocytes can act as an indicator for the progression of a disease and identifying proliferative lupus kidneys (Mansur et al., 2016).

### 3.2 Cytokines

After analyzing 1000 urine protein indicators from 30 patients with active lymphoblastic leukemia, Fava et al. (2020) discovered that patients with proliferative LN had higher IFN-γ levels. Combining single-cell transcriptomics from renal biopsies with urine proteomics demonstrated that IFN-γ caused chemokine gradients, which were mostly produced by myeloid, CD8^+^T, and natural killer cells entering the body. These urinary chemokine gradients showed a significant correlation with the quantity of CD8^+^ lymphocytes that infiltrate the kidney.

The urinary and plasma levels of interleukin-16 (IL-16) in patients with proliferative LN are higher than in those with mesangial proliferative LN and membranous nephropathy. Urinary IL-16’s diagnostic specificity for proliferative LN surpasses that of high-titer anti-double-stranded DNA and low complement C3 or C4 levels, although its sensitivity is slightly lower (Häyry et al., 2022). Another longitudinal study of 30 LN patients involved quantitative analysis of 1000 urinary proteins at four different time points—during renal biopsy and at 3, 6, and 12 months post-treatment—and single-cell transcriptomics of renal biopsy sections to evaluate intrarenal expression of candidate biomarkers. The study highlighted CD163, IL-16, and transforming growth factor β(TGF-β) among 237 urine biomarkers as reflecting renal inflammation activity (Fava et al., 2022). Single-cell RNA sequencing identified IL-16 as one of the most widely produced cytokines in most infiltrating immune cells in LN kidneys, with cells producing IL-16 located at key sites of renal injury. This positions IL-16 as a potential therapeutic target and urine biomarker (Fava et al., 2022). Additionally, uIL-17 levels were significantly higher in the proliferative LN group, with a diagnostic AUC of 0.717 and a threshold of 27.13 pg/mL, indicating its diagnostic efficacy for proliferative LN (25). A cohort study of 96 pediatric SLE patients demonstrated that elevated serum IL-18 levels at 6 months post-treatment were linked to adverse clinical outcomes, though there were no findings related to urinary IL-18 or renal pathology (Wu et al., 2016).

Colony-stimulating factor-1 (CSF-1), seen in the renal tubules, has been observed to enter the blood circulation in a lupus-susceptible mouse model, with its levels escalating alongside disease progression. Research indicates that elevated CSF-1 levels in serum or urine of LN patients correlate with increased CSF-1 expression in the kidney and heightened histopathological changes. Longitudinal patient follow-ups revealed that elevated serum or urine CSF-1 levels can prognosticate glomerular dysfunction and pre-clinical recurrence (Menke et al., 2015).

Those with active LN had significantly greater urinary tumor necrosis factor-related weak apoptosis inducer (uTWEAK) levels than those without LN, those with inactive renal disease, and healthy individuals (Salem et al., 2018). TWEAK’s sole receptor, fibro-blast growth factor-inducible type 14 (Fn14), is expressed in renal lamina propria cells, encompassing podocytes, thylakoid cells, tubular cells, and glomerular endothelial cells. Elevated uTWEAK levels corresponded with increased TWEAK expression in the kidney. uTWEAK levels also positively correlated with SLEDAI and r-SLEDAI scoring systems and peaked during nephrotic episodes, indicating that uTWEAK is a specific and sensitive biomarker for active LN detection (Chen et al., 2017).

A meta-analysis involving four LN diagnostic studies (276 patients) showed that urine TWEAK has extremely high diagnostic value for LN and LN activity. This study showed that the pooled sensitivity and specificity of uTWEAK for the diagnosis of LN were 0.55 (95% CI 0.47–0.63) and 0.92 (95% CI 0.86–0,96), respectively. The pooled sensitivity and specificity for the diagnosis of LN activity were 0.91 (95% CI 0.82–0.96) and 0.70 (95% CI 0.58–0.81), respectively (Wang et al., 2021). In another meta-analysis, subgroup analysis revealed that the pooled sensitivity, the diagnostic odds ratio (DOR) and AUC of TWEAK in predicting active LN were higher in patients with r-SLEDAI >4 than patients with r-SLEDAI >0. uTWEAK also revealed a higher pooled DOR than sTWEAK (Ma et al., 2021). Two systematic reviews have reached a consistent conclusion that uTWEAK is an auspicious biological marker for diagnosing LN (Guimarães et al., 2022).

The tumor necrosis factor family contains blymphocyte stimulator (BLyS), also referred to as B cell-activating factor, or BAFF. It forms the “BAFF system” with three receptors: transmembrane activator, BAFF receptor (BAFF-R), calcium-modulating cyclophilin ligand interactor (TACI), and B cell maturation antigen (BCMA), in addition to a proliferation-inducing ligand (April) (Sakai and Akkoyunlu, 2017). Patients with LN had urine samples tested for the BAFF system, most biomarkers expressed more frequently in classes III and IV LN than in class V LN. Histological activity indices and SLEDAI-2K were also correlated with the expression levels of uBAFF-R mRNA, indicating that the BAFF/April signaling factors in LN urine could be helpful biomarkers for the condition (Aguirre-Valencia et al., 2020).

Monocyte chemo-attractant protein-1 (MCP-1, CCL2) levels are notably elevated in urine samples from patients with active LN, correlating with histological features such as cell crescents, intracapillary hyperplasia, interstitial inflammation, glomerulosclerosis, interstitial fibrosis, and tubular atrophy. MCP-1 concentrations are significantly higher in diffuse proliferative LN compared to focal proliferative LN and membranous LN (Mohammed et al., 2014). Pérez-Arias, A. et al. discovered that urinary MCP-1 levels decreased in patients with long-term stable renal function 3 months post-LN treatment. Conversely, persistent elevated urinary MCP-1 levels during the first year post-treatment predicted renal function loss, indicating that urinary MCP-1 levels reflect not only LN histological activity and chronic lesions but also long-term renal function survival (Pérez-Arias et al., 2023). Two meta analyses evaluated the diagnostic accuracy of uMCP-1 in diagnosing LN and LN disease activity (Lee and Song, 2017; Xia et al., 2020). A total of seven original studies with 521 participants were included in one of the latest meta-analyses, 521 patients were included in 7 studies. The results showed that the pooled sensitivity was 0.89 (95% CI 0.86–0.93), the pooled specificity was 0.63 (95% CI 0.55–0.69), DORwas 19.4 (95% CI 7.24–51.96), and the summary ROC curve area under the curve (SROC-AUC)was 0.90 (Xia et al., 2020). System overview also prompts that MCP-1 seems to be superior to the conventional serological biomarkersused in the management of LN (Guimaraes et al., 2022).

CXCL10, or IFN-γinducible protein 10(IP-10), a chemokine released by IFN-γ stimulated endothelial cells, plays a role in autoimmune diseases. Its levels significantly correlate with renal activity scores, 24-h urine protein, and SLEDAI scores (Marie et al., 2014). A subgroup analysis in a clinical study involving 227 SLE patients revealed that urinary CXC chemokine ligand 4 (CXCL4) was significantly associated with histological activity scores in 68 patients who underwent both LN renal biopsies and baseline urine collection (Mok et al., 2018). Urinary fractalkine (CX3CL1, Fkn) and serum receptor for advanced glycation end product (RAGE) levels were increased in proliferative LN compared to non-proliferative cases, suggesting a potential link between fractalkine and RAGE concentrations and different pathological types of LN (Lan et al., 2016). While these studies indicate urinary chemokines have diagnostic and predictive value in LN, a single chemokine cannot yet be universally applied as a biomarker or potential therapeutic target. A meta-analysis showed that urine CXCL10 tended to be higher in patients with active-LN compared to non-active LN patients, but did not reach statistical significance (Puapatanakul et al., 2019).This is due to significant individual variations in LN urinary chemokines and variability in chemokine abundance (Klocke et al., 2017).

A globular protein called epidermal growth factor (EGF) is produced in the kidney, particularly in the glomerulus, Henle’s loop, and distal tubule, plays a critical role in renal health. Studies have demonstrated that urinary EGF/Cr levels are connected favorably with the estimated glomerular filtration rate (eGFR) at relapse and negatively correlated with histological chronicity indices observed in renal biopsies. These indices include glomerulosclerosis, interstitial fibrosis, and tubular atrophy (Segarra et al., 2019). Mejia-Vilet, J.M. et al. identified that urinary EGF values below 5.3 ng/mg serve as a cutoff for predicting the progression of LN to ESRD. Additionally, lower urinary EGF levels at the onset of LN and progressively decreasing EGF levels during treatment were connected to a poor long-term renal prognosis (Mejia-Vilet et al., 2021). Urinary transforming growth factor beta (uTGF-β1) and uIL-17 levels were much greater in the proliferative LN group compared to the control group. The AUC for uTGF-β1 was 0.665, with a cutoff value of 27.13 pg/mL, indicating its strong diagnostic potential for proliferative LN (Susianti et al., 2015). Activin A, a member of the transforming growth factor super-family, is created by macrophage infiltration. Animal studies have shown that Activin A is detectable in the urine of MRL/lpr mice at 16 weeks. The urinary levels of Activin A were significantly correlated with the number of perivascular inflammatory cell infiltrates, the number of crescentic glomeruli, and the percentage of positive fibrotic areas as indicated by Elastica-van-Gieson (EvG) staining. These results imply a function for infiltrating macrophage-derived Activin A in the development of renal damage in MRL/lpr mice. (Kadiombo et al., 2017).

### 3.3 Adhesive molecules

Patients with active LN were shown to have greater levels of vascular cell adhesion molecule 1 (VCAM-1, CD106) than those with inactive LN. This finding demonstrated a strong connection with histological activity indices in renal biopsies (Rahman, 2012). In a study examining the correlation of urinary VCAM-1 with nine renal histopathological manifestations, such as glomerular leukocyte infiltration, intracapillary proliferation, cellular crescents, fibrin-like necrosis, glomerulosclerosis, interstitial inflammation, fibro crescents, interstitial fibrosis, and tubular atrophy, urinary VCAM-1 was closely linked to fibro crescents. This association suggests that urinary VCAM-1 can be used as a non-invasive urinary biomarker (Soliman et al., 2017).

Urinary-activated leukocyte cell adhesion molecule (uALCAM) not only corresponds to SLE disease activity but also with the type of kidney damage pathology. Urinary ALCAM levels were shown to be considerably greater in class III and IV (proliferative) LN as opposed to class V LN in a Chinese investigation. These levels also showed a positive correlation with the activity index (AI), but not with the chronicity index (CI) (Ding et al., 2020). ALCAM, serving as a ligand for CD6, plays a part in T-cell activation and transport. Renal histopathology revealed CD6 was mainly expressed in T cells, and ALCAM levels were raised in the epithelial cells of renal tissue. Additionally, animal experiments demonstrated that ALCAM and CD6 are expressed in CD4^+^ T cells in SLE mouse models (Chalmers et al., 2022). The significance of the CD6/ALCAM pathway as a disease biomarker and possible target for therapy is highlighted by these findings, which point to urinary ALCAM as a potential biomarker for predicting renal pathogenic activity in LN.

### 3.4 Soluble leukocyte Markers/BCR

Soluble CD163 (sCD163), secreted by monocytes and macrophages, is emerging as a potential biomarker for diseases associated with macrophage hyper-activation. However, a recent study showed that CD163^+^dendritic cells (DC3) are also involved in LN and positively correlated with the severity of LN. The enumeration of renal DC3 holds potential as a valuable stratification feature for guiding LN patient treatment decisions in clinical practice (Chen et al., 2024).

In patients with active LN, urinary sCD163 (u-sCD163) levels are markedly elevated. A high prevalence of CD163-positive cells is observed in glomerular and acute tubulointerstitial lesions in LN-IV patients, with glomerular CD163-positive cells favorably connected with proteinuria and conversely correlated with estimated glomerular filtration rate. U-sCD163 aligns with histological activity indices in patients undergoing repeat renal biopsies (Li et al., 2015; Mejia-Vilet et al., 2020). Moreover, u-sCD163 is indicative of treatment response and prognosis in LN, decreasing progressively in complete and partial remitters, while remaining elevated in non-responders (Mejia-Vilet et al., 2020). Single-cell RNA sequencing analysis identifies macrophages as the primary CD163-expressing cells in LN kidneys, likely contributing to elevated u-sCD163 levels. U-sCD163 levels correlate closely with glomerular CD163^+^ cell counts, histological disease scores, and urinary monocyte chemotactic protein one level. Additionally, u-sCD163 is associated with SLEDAI, r-SLEDAI, renal pathological AI, fibrin-like necrosis, cell crescents, and interstitial inflammation in biopsied tissues. U-sCD163 can differentiate active LN from proliferative LN (Endo et al., 2016; Zhang et al., 2020), highlighting its potential as a biomarker of LN macrophage-enriched renal inflammation and its correlation with specific pathological changes. A strong predictor of full treatment response, CD163 was found to be the most significant urinary protein differentiator between responders and non-responders (median 1.8 pg/mgCr vs. 8.2 pg/mgCr, p = 4e-7) in LN patients in the Belimumab International Systemic Lupus Erythematosus-Lupus Nephritis trial (Weeding et al., 2022).

Integrin Mac-1 and CD16b, leukocyte surface markers, are associated with the pathophysiology of LN. Mac-1, with a distinct alpha subunit (CD11b) and a common beta2 subunit, is released from specific leukocyte sub-populations along with CD16b under inflammatory conditions, including glomerulonephritis. In individuals with biopsy-proven glomerular illness, including LN, urine CD11b and CD16b levels are correlated with histopathological activity, and urinary CD11b is predictive of proliferative LN. Urinary CD11b declines after treatment and is correlated with glomerular leukocyte counts and overall histopathological activity as *in vitro* studies indicate that leukocyte activation and migration are necessary for CD11b shedding in urine, indicating that urinary CD11b may serve as a biomarker for evaluating the histopathological activity of LN, especially in glomerular leukocyte aggregation (Kitagawa et al., 2019).

Immunoglobulin-binding protein 1 (IGBP1), is connected to the B-cell receptor (BCR) domain, is detectable in LN renal tubules. Lee, E. J., et al. found that IGBP1, primarily expressed in CD14^+^ cells in LN patients, has significantly elevated urinary levels correlating with clinical and histological activity indices (Lee et al., 2019).

### 3.5 Antibodies, light chain fragments and complement degradation products

Serum anti-C1q antibodies and urinary ceruloplasmin are correlated with renal biopsy activity in SLE patients, while MCP-1 is linked to chronic damage. These biomarkers may be used in conjunction with other techniques to improve the categorization of LN patients, as they have been validated in a larger multi-ethnic population (Gómez-Puerta et al., 2018; Urrego-Callejas et al., 2021). Additionally, antibodies such as anti-RNA polymeraseI, anti-dsDNA, anti-La, and anti-ribosomal P have been found at levels associated with disease activity in the urine of individuals with LN.

A study revealed that urinary free light chains (FLCs) were considerably in proliferative LN as opposed to nonproliferative subgroups. Urinary λ-FLC levels showed a significant correlation with the urinary protein/creatinine in class III/IV LN subgroups. Furthermore, CD19^—^/CD138^+^ cell counts of secretory LCs in renal specimens were greater in class III/IV LN subgroups compared to non-class III/IV subgroups. Total urinary FLC levels correlated with the number of CD138^+^cells in the kidney, indicating a relationship between the degree of renal plasma cell infiltration and urinary FLC levels, thus suggesting their potential as useful biomarkers in proliferative LN (Hanaoka et al., 2013).

Complement degradation products have been used for monitoring disease activity.Previous studies have shown that urinary C3d excretion is increased in active LN patients and was significantly more as compared to inactive LN and non-renal disease. An increase in urinary C3d excretion in active LN patients, which is significantly higher than in non active LN and non renal diseases in a longitudinal study (Ganguly et al., 2020a).

### 3.6 Proteins and small molecule peptides

Urinary neutrophil gelatinase-associated lipocalin (uNGAL) and MCP-1, alongside anti-C1q antibodies, have proven useful as indicators for identifying the involvement of the kidney and distinguishing activeLNamong Latin-American SLE patients (Gómez-Puerta et al., 2018). According to a study, uNGAL levels were substantially higher in the proliferative LN group compared to the control group. uNGAL demonstrated good diagnostic value for proliferative LN and strongly connected with activity and prolonged indices, positioning NGAL as a potential indicator in proliferative LN (Susianti et al., 2015). Two meta-analyses evaluated the role of NGAL in LN, with the latest review including 19 articles (Gao et al., 2020; Fang et al., 2015). These two meta-analysesidentified results in the same direction for the diagnostic accuracy of u-NGAL. In a systematic review evaluating the diagnostic accuracy of novel biomarkers for LN, three biomarkers,uMCP-1, uTWEAK, and uNGAL have outstanding value in the diagnosis of LN (Guimaraes et al., 2022).

Kidney injury molecule-1 (Kim-1), expressed in damaged renal tubules, shows that urinary Kim-1 levels significantly correlate with Kim-1 expression in renal tissues. uKim-1 levels assist in determining LN activity and can assess Kim-1 expression in renal biopsies. This correlation helps in predicting pathological changes such as renal injury, persistent glomerulonephritis, tubulointerstitial inflammation, and tubular atrophy in LN histopathology (Nozaki et al., 2014).

Urinary galectin-3 binding protein (uG3BP) in individuals with active LN effectively distinguishes proliferative LN and positively correlates with the AI and hyaline deposition scores. Notably, in patients with LN and a 24-h urine protein exceeding 3.0 g, uG3BP levels are greater in regenerative LN compared to membranous LN. This suggests that uG3BP is linked with active histological changes and proliferative LN, offering a viable alternative biomarker when renal biopsy is not feasible (Ding et al., 2022).

Neuraminidase 1 (NEU1), pivotal in the degradation pathway of glycans, was discovered to be highly expressed in the kidneys of patients with proliferative LN through proteomic analysis. NEU1 co-localizes with podocytes, thylakoid cells, endothelial cells, and tubular cells. Significantly, NEU1 representation in the tubular interstitial region of LN patients with a CI greater than 1 was markedly higher compared to those with a CI less than 1 or healthy controls. Hence, elevated renal NEU1 expression is found to be a separate risk factor for the prognosis of the kidneys. (Mao et al., 2021).

Urinary high-mobility group box one protein (HMGB-1) levels are increased in patients with active LN, patients with class V LN had higher levels than those with proliferative and mixed types. However, further evaluation in a larger lupus population is necessary (Jog et al., 2016).Catalina Burbano et al. observed that urinary HMGB1^+^ microparticles differed between patients with active and inactive LN, suggesting that these microparticles in urine are indicative of renal among individuals with systemic lupus erythematosus (Burbano et al., 2019).

Urinary alpha-1-acid glycoprotein (ORM1) has emerged as a potential early detection signal of LN, even before the onset of massive proteinuria. Additionally, urinary hemoglobin subunit δ (HBD) accurately differentiates between proliferative and non-proliferative LN and correlates with activity indices, which is particularly beneficial when renal biopsy is not feasible (Kwon et al., 2021).

An early elevation and subsequent increase of urinary SerpinA3 (uSerpinA3) were noted in a rat model transitioning from acute kidney injury (AKI) to chronic kidney disease (CKD). In CKD patients with focal and segmental glomerulosclerosis, ANCA-associated vasculitis, and proliferative grade III and IV LN, uSerpinA3 levels were independently positively connected with renal fibrosis. However, in patients with grade V LN, uSerpinA3 levels did not significantly differ from those in healthy volunteers. This suggests that uSerpinA3 can detect renal fibrosis and inflammation, particularly useful for early detection of the transition from AKI to CKD, and can differentiate between LN grades III/IV and V (Sánchez-Navarro et al., 2019). Another study indicated that uSerpinA3 levels in grade IV LN were greater than those in grade III, correlating with histological activity indices. Additionally, uSerpA3 significantly decreased in treatment responders at 6 months while remaining consistently elevated in non-responders. Thus, uSerpinA3 serves as an early indicator of renal inflammation and a predictor of clinical response to therapy in patients with proliferative LN (Martinez-Rojas et al., 2022).

Research revealed significantly elevated levels of serum pentraxin-3 (PTX3) in patients with active LN, showing a significant relationship between PTX3 levels and renal pathology index scores, particularly interstitial inflammation in active nephritis. PTX3 also correlated with several renal tubular interstitial lesion indicators, including uKim-1 and uNGAL, suggesting its importance as an indicator of illness progression and renal tubular biomarkers of interstitial injury (Pang et al., 2016).

The transmembrane receptor neuropilin 1 (NRP-1), highly expressed in mesangial cells, was found to be elevated in renal biopsies of LN patients. According to a study, individuals with active LN had greater urine and renal tissue NRP-1 levels, and those who showed clinical response had even higher levels. NRP-1 also surfaced as an independent marker of clinical response. These findings suggest that NRP-1 can function as a biomarker for early prognosis for LN (Torres-Salido et al., 2019).

Excessive accumulation of extracellular matrix (ECM) in the renal tubular interstitium is a critical factor in chronic kidney damage in LN and plays a pivotal role in disease progression to renal failure. Novel biomarkers for ECM renovating are now being recognized as indicators of renal fibrosis, tubular atrophy, and disease activity in LN patients. A study identified a total of 70 collagen and 230 non-collagen peptides with significant alterations between LN patients and healthy SLE volunteers without renal damage, suggesting these changes may represent risky procedures involving ECM transformation in LN kidneys (Wei et al., 2017). Another study revealed that serum interstitial collagen (PRO-C6) and urinary collagen type III (C3M), among other biomarkers of interstitial collagen conversion, associated with histological markers of interstitial fibrosis, tubular atrophy, and monocyte infiltration. These findings indicate the potential for noninvasive urinary collagen turnover biomarkers to be developed for LN diagnosis and monitoring renal disease progression (Genovese et al., 2021).

### 3.7 Metabolomics

Metabolomics has shown promise in identifying potential markers for pathological changes in different types of LN. A study utilizing 1H-nuclear magnetic resonance (1H-NMR) metabolomics compared urinary metabolism in 14 LN patients, 10 SLE patients, and 11 healthy controls (HC). Three metabolites—β-alanine, 2,2-dimethyl succinic acid, and 3,4-dihydroxyphenylacetaldehyde—were recognized as elements of a diagnostic model for LN with an AUC of 0.89, a sensitivity of 81%, and a specificity of 100%. Pathway analysis pointed to nicotinate and nicotinamide metabolism as the most significantly altered pathways in LN (Kalantari et al., 2019)

In a study comprising discovery (18 HCs and 20 LN patients) and validation cohorts (53 HCs and 64 LN patients), the pyridoxine (Pic) to tryptophan (Trp) ratio (Pic/Trp) was identified as an excellent contender for LN diagnosis and categorization of proliferative and membranous LN. This ratio shows plenty of promising as an alternative non-invasive biomarker for LN diagnosis (Anekthanakul et al., 2021).

Urinary citrate, a marker of reduced aerobic glycolysis and oxidative phosphorylation, may also utilize as a non-invasive biomarker for diagnosing and monitoring LN therapy responses. A study comparing urinary citrate and acetate excretion levels in biopsy-proven LN patients before and 6 months after cyclophosphamide induction therapy found that median urinary citrate and creatinine levels were substantially reduced in LN patients than in HCs and notably higher 6 months post-therapy. The AUC was 0.9136 for urinary citrate and 0.6883 for urinary acetate in differentiating LN from HCs. Urinary acetate levels positively connected with SLEDAI, whereas urinary citrate levels positively correlated with C3 and negatively with urinary protein/creatinine (Ganguly et al., 2020b).

### 3.8 Urine exosomes and ribonucleic acid

Exosomes, which are micro-vesicles secreted by epithelial cells, are becoming more and more acknowledged as a prime source of indicators for renal damage and dysfunction. A study screened for tRNA derived small noncoding RNAs (tsRNAs) in the urine exosomes of LN and non-LN patients during the training phaseand validation phase.The results showed that the levels ofthe urinary exosomestRF3-Ile-AAT-1 and tiRNA5-Lys-CTT-1 were upregulated, which has high diagnostic value for LN and active LN (Chen et al., 2023).Studies indicate that miR-29c expression in the urinary exosomes of LN patients is inversely correlated with the renal histological CI and glomerulosclerosis, but not with renal function. This suggests its possibility as a novel noninvasive marker for early fibrosis progression in LN patients (Solé et al., 2015; Cardenas-Gonzalez et al., 2017; Perez-Hernandez et al., 2021). Research also showed a downregulation of miR-3201 and miR-1273e among 2402 urinary microRNAs (miRNAs) in LN patients, correlating with intracapillary glomerular inflammation (Cardenas-Gonzalez et al., 2017).Exosomal miR-146a is negatively correlated with circulating C3 and C4 complement components, proteinuria, and chronic histological features. Further *in vitro* tests showed that miR-146a exerts a safeguarding effect by negatively regulating inflammation through the inhibition of IRAK1 and TRAF6. The progression of renal fibrosis in LN is closely linked with the miR-146a-TRAF6 axis (Perez-Hernandez et al., 2021). Proliferative LN kidneys and urine show reduced levels of miRNAs miR-26a and miR-30b, which directly control the cell cycle of thylakoid cells. The expression of these miRNAs is influenced by HER-2, over-expressed in the kidneys and urine of NZM2410 mice and LN patients. Therefore, HER-2, miR-26a, and miR-30b emerge as probable markers for LN, and inhibiting HER-2 could be an advantageous approach to lessen proliferation of cells and damage in this condition (Costa-Reis et al., 2015).

Key transcription factors of T cells in urine may reflect the intrinsic characteristics of patients prone to T cell activation. Monitoring urinary sediment mRNA levels of T-bet (key transcription factor of type 1 T helper cells), GATA-3 (key transcription factor of type 2 T helper cells), and FOXP3 (key transcription factor of regulatory T cells) could provide early indicators of disease flares in lupus patients (Szeto, 2017). While urinary mRNA quantification holds potential for clinical research in SLE, current studies in this area are finite and predominantly small-scale. Therefore, more extensive sample studies are necessary to confirm its usefulness in the risk assessment and monitoring of LN patients.

### 3.9 Urinary biological marker assessment model

A study has demonstrated the powerful function of low-abundance biomarker panels and machine learning algorithms for predicting 1-year outcomes for the first time in predicting treatment response in LN. The predictive model combining new urine biomarkers and clinical biomarkers developed has more clinical predictive ability than biomarkers developed solely using traditional clinical biomarkers.It is interesting that the most predictive models are the combination of osteoprotegerin (OPG), IL-2Rα, urinary protein/creatinine, IL-8, and TWEAK, while race, anti dsDNA antibodies, and induction medication were not significant contributors to the model (Wolf et al., 2016). A subsequent study using machine learning also reached similar conclusions (Ayoub et al., 2022)

The Renal Activity Index for Lupus Nephritis (RAIL), comprising six urinary proteins—NGAL, MCP-1, ceruloplasmin, adiponectin, hemopexin, and Kim1—serves as an ideal predictor of renal activity in LN among children (Brunner et al., 2016). Gulati G. et al. demonstrated that RAIL is equally effective for predicting adult LN activity. The results of a logistic regression analysis showed that the RAIL scores of these six urine-based biological markers were strong predictors of adult LN activity, with an AUC of 0.88 (Gulati et al., 2017).

The expression levels of biomarkers, including serum and urinary IFN-γ, CXCL16, and soluble urokinase plasminogen activator receptor (uPAR), were found to be notably greater in LN patients than in normal control subjects.These levels associated with the activity of pathological lesions in renal tissues, effectively reflecting disease activity, renal injury, and pathological lesions in SLE patients (Wen et al., 2018).

A study showed that the integrated model of uMCP-1 and uTWEAK outperforms traditional models, including β2-microglobulin and serum C3, C4, creatinine (Cr), blood urea nitrogen (BUN), and cystatin C, in evaluating renal SLEDAI and biopsy activity index (BAI) score ≥7. The predictive model, integrating MCP-1 and uTWEAK, demonstrated a high correlation coefficient with renal damage and a larger area under the ROC curve (Dong et al., 2018).This finding suggests that the combined model of uMCP-1 and uTWEAK serves as a reliable marker for rapidly identifying severe renal damage.

uCystatinC, uKIM-1, uVCAM-1, and urinary vitamin D-binding protein (uVDBP) were discovered to have a positive connection with the renal nephrogenic AI, while uVCAM-1 also showed a positive correlation with the CI. The combined assay of uVCAM-1 can effectively distinguish proliferative LN from membranous LN. The combination of uVCAM-1, uCystatinC, and uKim-1 serves as a reliable set of urinary biomarkers reflecting renal pathology (Liu et al., 2020).

A study involving Luminex analysis of 128 analytes in urine indicated a significant increase in 44 analytes in patients with active LN. This group included several unique proteins like tissue inhibitors of metalloproteinases (TIMPs), plasminogen activator inhibitor-1 (PAI-1), platelet factor 4 (PF4), von Willebrand factor (vWF), and IL-15, in addition to the acknowledged candidate LN biomarkers (such as lipocalin, sVCAM-1, and IL-6). These analytes showed a significant (>4-fold) distinction between active LN and non-LN patients and returned to baseline levels in LN patients in remission. These findings were verified in the validation cohort. The identified proteins demonstrated a superior ability to differentiate active LN from non-LN compared to several biomarkers previously documented (Landolt-Marticorena et al., 2016). Subsequent studies found that lipocalin, MCP-1, sVCAM-1, and PF4 had greater predictive value for proliferative active LN (Whittall-Garcia et al., 2022).

The LN chronicity index was associated with urinary exosomal miR-21, miR-150, and miR-29c. The combination of these markers predicted a higher chance of the advancement of ESRD. Pathway analysis identified vascular endothelial growth factor A and specificity protein 1 (SP1) as shared target genes for these miRNAs. Immunohistochemistry of LN kidney biopsies showed noteworthy increases in type I collagen alpha one chain (COL1A1) and type IV collagen alpha one chain (COL4A1) associated with renal chronicity. Further *in vitro* tests indicated that these miRNA combinations enhanced pre-fibrotic molecules via the SP1 and Smad3/TGFβ pathways, promoting renal fibrosis. This suggests that urinary exosomal multi-marker combinations, including miR-21, miR-150, and miR-29c, offer a non-surgical method to identify early renal fibrosis and forecast the course of LN disease (Solé et al., 2019). Table 1 summarizes the correlation between urinary biological markers and renal pathology.

**TABLE 1:** Urinary biological markers related to the pathology of lupus nephritis.

Types of urinary biomarkers	Correlation with renal pathology
Cell Components	
CD4^+^T	Associated with the severity and pathological type of LN (Scott et al., 2016; Mesquita et al., 2017)
PS/PC	Associated with proliferative nephritis and renal function loss (Scott et al., 2016)
Macrophage	Associated withchronic glomerular index, tubular atrophy, and interstitial fibrosis (Kim et al., 2020)
CD14^+^cell	Useful in distinguishing different categories of proliferative LN (Abdelati et al., 2021)
Podocyte	Associated with proliferative LN (Wang et al., 2015; Mansur et al., 2016)
Cytokines	
IFN-γ	Associated with renal CD8+lymphocyte infiltration (Fava et al., 2020)
Interleukin	IL-16 is associated with the severity of renal inflammatory cell infiltration (Häyry et al., 2022; Fava et al., 2022). IL-17 levels correlate with proliferative LN (Susianti et al., 2015), while IL-18 is linked to a poor prognosis in these patients (Wu et al., 2016)
CSF-1	A key predictor of glomerular dysfunction and recurrence in LN (Menke et al., 2015)
TNF family	uTWEAK is associated with the renal SLEDAI activity score, reflecting its involvement in disease activity (Salem et al., 2018; Chen et al., 2017). uBLyS correlates with proliferative LN (Sakai and Akkoyunlu, 2017; Aguirre-Valencia et al., 2020)
Chemokine	CCL2 is associated with renal activity, chronic lesions, and the long-term survival of renal function, signifying its role in both disease activity and prognosis (Mohammed et al., 2014; Pérez-Arias et al., 2023). CXCL10 mirrors histological inflammation in LN (Marie et al., 2014), while CXCL4 significantly correlates with histological activity scores (Mok et al., 2018). CX3CL1 is linked to proliferative LN (Lan et al., 2016)
Growth Factor	The level of EGF inversely correlates with the chronic pathological index of the kidney, suggesting its role in renal tissue remodeling (Segarra et al., 2019; Mejia‐Vilet et al., 2021). TGF-β1 is related to proliferative LN, highlighting its influence on disease severity (Susianti et al., 2015). Activin A relates to vasculitis cell infiltration, the number of crescentic glomeruli, and fibrosis (Kadiombo et al., 2017)
Adhesion Molecule	
VCAM-1	Shows a significant correlation with fibrous crescents (Rahman, 2012; Soliman et al., 2017)
ALCAM	Exhibits a positive correlation with the AI (Ding et al., 2020; Chalmers et al., 2022)
Soluble Leukocyte Markers/BCR	
sCD163	Reflects the histological inflammation index of LN, predicts treatment efficacy and prognosis (Li et al., 2015; Mejia-Vilet et al., 2020), and differentiates proliferative LN and differentiate proliferative LN (Endo et al., 2016; Zhang et al., 2020).It is also predictive of treatment response (Weeding et al., 2022)
sCD11b	Associated with active lesions, particularly glomerular leukocyte aggregation (Kitagawa et al., 2019)
IGBP1	Associated with clinical and histopathological active lesions (Lee et al., 2019)
Antibodies, light chain fragments and complement degradation products	
Anti-C1q	Correlates with renal pathological activity (Gómez-Puerta et al., 2018; Urrego-Callejas et al., 2021)
Free Light Chain C3d	Indicates renal plasma cell infiltration intensity, associated with proliferative LN (Hanaoka et al., 2013) As a biomarker of disease activity and treatment response (Ganguly et al., 2020a)
Proteins and small molecule peptides	
NGAL	Identifies renal involvement and distinguishes active LN (Gómez-Puerta et al., 2018).Related to active LN and proliferative pathology (Susianti et al., 2015)
MCP1	Identifies renal involvement and discriminates active LN (Gómez-Puerta et al., 2018)
KIM 1	Associated with pathological changes such as renal injury, persistent glomerulonephritis, tubulointerstitial inflammation, and tubular atrophy in LN (Nozaki et al., 2014)
G3BP	Linked to proliferative nephritis and AI (Ding et al., 2022)
NEU1	Associated with proliferative pathology and LN chronic index (Mao et al., 2021)
HMGB-1	Associated with V-LN (Jog et al., 2016).HMGB1+ microparticles connected to active LN (Burbano et al., 2019)
ORM1	Differentiates proliferative LN and correlates with AI (Kwon et al., 2021)
SerpinA	Early nephritis indicators, predictive of efficacy in proliferative LN (Sánchez-Navarro et al., 2019; Martínez-Rojas et al., 2022)
PTX-3	Associated with active lesions and renal tubulointerstitial injury (Pang et al., 2016)
NRP-1	Indicative of active nephritis and serves as early prognostic biomarkers (Torres-Salido et al., 2019)
**ECM**	Associated with interstitial fibrosis and tubular atrophy in LN patients (Wei et al., 2017; Genovese et al., 2021)
Metabolomics	
β-alanine 2,2-dimethylsucssinic acid,3,4-Dihydroxyphenylacetaldehyde	For diagnosing LN (Kalantari et al., 2019)
Pic/Trp	Specific biomarkers for membranous LN (Anekthanakul et al., 2021)
citrate	Useful for diagnosis and monitoring treatment response in LN (Ganguly et al., 2020b)
Exosomes/RNA	
tsRNAs miR-29c	For diagnosing LN (Chen et al., 2023) Associated with early fibrosis (Solé et al., 2015)
miR-3201, miR-1273e	Associated with inflammation in glomerular capillaries (Cardenas-Gonzalez et al., 2017)
miR-146a	Associated with Chronic Nephritis Index (Perez-Hernandez et al., 2021)
miR-26a, miR-30b	Associated with proliferative lesions (Costa-Reis et al., 2015)
T-bet, GATA-3, FOXP3 mRNA	Provide early clues for lupus flare in patients (Szeto, 2017)
Urinary Biological Marker Assessment Model	
RAIL	Predictive of Renal Activity in LN (Brunner et al., 2016; Gulati et al., 2017)
**IFN-γ, CXCL16, uPAR**	Related to active pathological changes in LN (Wen et al., 2018)
uMCP-1, uTWEAK	Closely related to renal SLEDAI score, biopsy activity index (BAI), and biopsy chronic index (Dong et al., 2018)
uCystatinC, uKIM-1, uVCAM-1, uVDBP	Positively correlated with AI, distinguishing proliferative LN (Liu et al., 2020)
Adiponectin, MCP-1, sVCAM-1, PF4	Indicative of proliferative active LN (Landolt-Marticorena et al., 2016; Whittall-Garcia et al., 2022)
miR-21, miR-15, miR-29c	Related to LN chronic index (CI), serve as a combination for early renal fibrosis and prediction of LN disease progression (Solé et al., 2019)

## 4 Conclusion

Urine, as a non-invasive source for monitoring renal inflammatory status in SLE patients, is garnering increasing interest. Analyzing LN biomarkers from a pathological standpoint holds greater clinical significance. The integration of biological markers found in LN urine with pathological types, activity levels, chronic fibrosis, and other pathological characteristics offers a potential alternative to invasive kidney biopsies.

However, most urine biomarkers are confined to cross-sectional studies, and only a few have been evaluated in longitudinal studies and independently validated (Capecchi et al., 2020). Although some high-quality systematic reviews highlight the significance of some urine biomarkers, standardized methods and composing LN diagnostic panels in cohort studies and clinical diagnostic randomized trials are still needed.Currently, advancements in various high-throughput technologies and omics research are fueling the discovery of promising urinary biomarkers. These novel urine biomarkers, or combined models, are poised to significantly enhance the diagnosis, monitoring, and prognosis of LN patients in the immediate future.

## Figures and Tables

**Table:** 

**LN**	Lupus nephritis
**SLE**	Systemic lupus erythematosus
**ESRD**	end-stagerenal disease
**ISN/RPS**	International Society of Nephrology/Renal Pathology Society
**TMA**	thrombotic microangiopathies
**NIH**	National Institutes of Health
**PB**	Plasmablasts
**PC**	plasma cells
**IL-6**	interleukin-16
**CSF-1**	Colony-stimulating factor-1
**uTWEAK**	urinary tumor necrosis factor-related weak apoptosis inducer
**Fn14**	factor-inducible type 14
**SLEDAI**	SLE Disease Activity Index
**r-SLEDAI**	renal SLEDA
**BLyS**	Blymphocyte stimulator
**TGF-β**	transforming growth factor β
**April**	a proliferation-inducing ligand
**TACI**	transmembrane activator and calcium-modulating cyclophilin ligand interactor
**BCMA**	B cell maturation antigen
**MCP-1**	Monocyte chemoattractant protein-1
**DOR**	diagnostic odds ratio
**SROC-AUC**	summary ROC curve area under the curve
**RAGE**	receptor for advanced glycation end product
**EGF**	epidermal growth factor
**VCAM-1**	Vascular cell adhesion molecule 1
**uALCAM**	urinary-activated leukocyte cell adhesion molecule
**AI**	activity index
**IGBP1**	Immunoglobulin binding protein 1
**FLCs**	free light chains
**Kim-1**	kidney injury molecule-1
**NGAL**	neutrophil gelatinase-associated lipocalin
**uG3BP**	urinary galectin-3 binding protein
**CI**	chronicity index
**HMGB-1**	High-mobility group box one protein
**ORM1**	alpha-1-acid glycoprotein
**AKI**	acute kidney injury
**CKD**	chronic kidney disease
**PTX3**	pentraxin-3
**NRP-1**	neuropilin 1
**ECM**	extracellular matrix
**1H-NMR**	1H-nuclear magnetic resonance
**pyridoxine/tryptophan**	Pic/Trp
**RAIL**	Renal Activity Index for Lupus Nephritis
**uPAR**	urokinase plasminogen activator receptor
**BAI**	biopsy activity index
**uVDBP**	urinary vitamin D-binding proteinu
**PF4**	Platelet factor 4
**SP1**	specificity protein 1
**COL1 A1**	type I collagen alpha one chain
**COL4A1**	type IV collagen alpha one chain
**tsRNA**	tRNA derived small noncoding RNAs
**AUC**	Area Under the Curve
**OPG**	osteoprotegerin
**IL-2Rα**	interleukin −2 receptor α
